# Cytogenetic Analysis of Seven Species of Gekkonid and Phyllodactylid Geckos

**DOI:** 10.3390/genes14010178

**Published:** 2023-01-09

**Authors:** Gabriela Chrostek, Aleksandra Domaradzka, Alona Yurchenko, Lukáš Kratochvíl, Sofia Mazzoleni, Michail Rovatsos

**Affiliations:** Department of Ecology, Faculty of Science, Charles University, 12844 Prague, Czech Republic

**Keywords:** C-banding, FISH, Gekkota, heterochromatin, karyotype, rDNA, sex chromosomes, telomeres

## Abstract

Geckos (Gekkota), the species-rich clade of reptiles with more than 2200 currently recognized species, demonstrate a remarkable variability in diploid chromosome numbers (2n = 16–48) and mode of sex determination. However, only a small fraction of gekkotan species have been studied with cytogenetic methods. Here, we applied both conventional (karyotype reconstruction and C-banding) and molecular (fluorescence in situ hybridization with probes for rDNA loci and telomeric repeats) cytogenetic analyses in seven species of geckos, namely *Blaesodactylus boivini*, *Chondrodactylus laevigatus*, *Gekko badenii*, *Gekko* cf. *lionotum*, *Hemidactylus sahgali*, *Homopholis wahlbergii* (Gekkonidae) and *Ptyodactylus togoensis* (Phyllodactylidae), in order to provide further insights into the evolution of karyotypes in geckos. Our analysis revealed the presence of interstitial telomeric repeats in four species, but we were not able to conclude if they are remnants of previous chromosome rearrangements or were formed by an accumulation of telomeric-like satellite motifs. Even though sex chromosomes were previously identified in several species from the genera *Hemidactylus* and *Gekko* by cytogenetic and/or genomic methods, they were not detected by us in any examined species. Our examined species either have poorly differentiated sex chromosomes or, possibly, environmental sex determination. Future studies should explore the effect of temperature and conduct genome-wide analyses in order to identify the mode of sex determination in these species.

## 1. Introduction

Geckos (Gekkota) are the highly diversified group of squamates reptiles, with nearly 2200 currently recognized species distributed in warmer climates across the globe [1]. They represent an old radiation, with a basal split dated by some authors even to 180 Ma [2]. Geckos are currently categorized into seven families: Carphodactylidae, Diplodactylidae, Eublepharidae, Gekkonidae, Phyllodactylidae, Pygopodidae, and Sphaerodactylidae [1,3,4]. Among them, the family Gekkonidae is the most species-rich, including almost 70% of the extant species of geckos [1]. However, only a small fraction of gecko diversity has been cytogenetically analyzed so far.

A large variability in karyotypes has been encountered in geckos, both in terms of chromosome morphology and diploid numbers, which range from 2n = 16 in *Gonadotes taniae* [5] to 2n = 48 in *Cyrtodactylus consobrinus* [6]. In contrast to the majority of the sauropsids (i.e., reptiles and birds), geckos either lack or have just a few microchromosomes [7]. The Carphodactylidae, Diplodactylidae, and Pygopodidae group (Pygopodoidea) almost exclusively share a karyotype of 2n = 38, while the situation is more variable in the remaining families (Gekkonoidea group), despite the presence of a trend for mainly lower diploid numbers in the families Sphaerodactylidae and Eublepharidae, and higher in Phyllodactylidae and Gekkonidae [7,8,9,10,11,12].

Sex chromosomes, when present, represent a further source of variability. Indeed, both male and female heterogamety is present, and in a few cases, even multiple sex chromosomes have been recorded in geckos [9,13,14,15,16,17,18,19,20,21,22]. In addition, some lineages of geckos, such as members of the genera *Phelsuma* and *Eublepharis*, lack sex chromosomes, and the sex in these species is determined by environmental factors (Environmental Sex Determination—ESD), most commonly temperature (Temperature-dependent Sex Determination—TSD) [3,23,24].

In the present study, we cytogenetically examined seven species of geckos, namely *Blaesodactylus boivini, Chondrodactylus laevigatus, Gekko badenii, Gekko* cf. *lionotum, Hemidactylus sahgali, Homopholis wahlbergii* (Gekkonidae), and *Ptyodactylus togoensis* (Phyllodactylidae) in the attempt to add new pieces to the complex puzzle of karyotype evolution in geckos. We performed both conventional (karyotype reconstruction and C-banding) and molecular (fluorescence in situ hybridization with probes for rDNA loci and telomeric repeats) cytogenetic analyses in order to provide further insight into the evolution of their karyotypes.

## 2. Materials and Methods

### 2.1. Samples Collection

We collected blood samples from both sexes of seven species of geckos, namely *Blaesodactylus boivinii, Chondrodactylus laevigatus, Gekko badenii*, *Gekko* cf. *lionotum*, *Hemidactylus sahgali, Homopholis wahlbergii* (Gekkonidae), and *Ptyodactylus togoensis* (Phyllodactylidae). All geckos originated from legal imports for the pet trade, and species identification was based on external morphology. Geckos can easily shed their tails by autotomy; therefore, we did not collect blood samples from the caudal vein but instead from the brachial vein of the front legs using an insulin-type syringe with 50 μL of heparin solution (5000 IU/mL; Zentiva, Prague, Czech Republic). A detailed list of collected samples is presented in Table 1.

### 2.2. Chromosome Preparation and Staining

Chromosome suspensions were obtained by whole blood cell cultures [25]. The medium used for cultures consisted of 90 mL D-MEM cell culture medium (without glucose, L-glutamine, and sodium pyruvate; GIBCO, Carlsbad, CA, USA), 10 mL of fetal bovine serum (GIBCO, Carlsbad, CA, USA), 3 mL of phytohemagglutinin M (GIBCO, Carlsbad, CA, USA), 1 mL of penicillin/streptomycin solution (10,000 units/mL; GIBCO, Carlsbad, CA, USA), 1 mL L-glutamine solution (200 mM; Sigma-Aldrich, St. Louis, MO, USA), and 1 mL lipopolysaccharide solution (10 mg/mL; Sigma-Aldrich, St. Louis, MO, USA). An amount of 100–200 μL of blood were added to 5 mL of fresh medium and incubated for one week at 30 °C. After the incubation period, the mitotic cycle was arrested in metaphase by adding 35 μL of colcemid to each sample. Following an incubation period of 3.5 h at 30 °C, the samples were centrifuged at 1200 RPM for 10 min at room temperature and then incubated with 0.075 M of prewarmed KCl for 10 min at 37 °C, followed by three rounds of fixation with a 3:1 methanol:acetic acid solution.

Chromosome suspensions were dropped onto slides and stained with 6% Giemsa to visualize chromosomes for karyotype reconstruction.

We applied a C-banding stain to analyze constitutive heterochromatin, following the standard protocol of [26], with small modifications. In detail, the slides were incubated in 0.2N HCl for 20–25 min, treated with an oversaturated barium hydroxide solution for 5–10 min at 45 °C, and incubated with saline-sodium citrate buffer (2xSSC) for 1 h at 60 °C. The slides were stained with DAPI for microscope visualization.

### 2.3. *In Situ* Fluorescence Hybridization with Telomeric and 18S/28S rDNA Loci Probes

Fluorescence in situ hybridization (FISH) was conducted to examine the distribution of the telomeric repeats (TTAGGG)_n_ and 18S/28S rDNA loci. The telomeric (TTAGGG)_n_ probe was prepared by PCR without a DNA template [27], and the rDNA probe was prepared by nick translation using the pDmr.a 51#1 plasmid with an 11.5 kb insert encoding the 18S and 28S ribosomal units of *Drosophila melanogaster* [28]. Both probes were labeled with dUTP-biotin (Roche Diagnostics, Basel, Switzerland).

Slides with chromosome suspensions were washed in 2xSSC for 5 min and incubated subsequently with RNase A (100 ug/mL) for 1 h and 0.01% pepsin solution for 10 min, with intermediate washes in 2xSSC, three times for 5 min each. After incubation, the slides were washed three times in 1xPBS and incubated in a 1% formaldehyde solution for 10 min. Then, slides were washed in phosphate buffered saline (1xPBS) for 5 min and dehydrated, respectively, in 70%, 85%, and 100% ethanol for 5 min each. Dried slides were denatured in 70% formamide/4xSSC for 2 min at 70 °C, washed in 2xSSC for 5 min and dehydrated in the ethanol series again. In parallel, the probe was incubated for 6 min at 73 °C and then at −20 °C for 10 min. After this, 10 μL of the probe was applied to each slide and incubated overnight in a wet chamber at 37 °C.

After incubation, post-hybridization washes and signal detection were performed. Slides were washed in 2xSSC, three times in 50% formamide in 2xSSC at 37 °C, in 2xSSC twice, and once in 4xSSC/0.05% Tween20 (Sigma-Aldrich, St. Louis, MO, USA), each for 5 min. The next step was adding 4xSSC/5% blocking reagent (Roche Diagnostics, Basel, Switzerland) and incubating for 30 min at 37 °C. After incubation, slides were briefly washed in 4xSSC/0.05% Tween 20. The hybridization signal was enhanced with three subsequent incubations of avidin-FITC (5 ng in 100 mL of 4xSSC/5% blocking reagent; Vector Laboratories, Burlingame, CA, USA) and two intermediate incubations of biotinylated anti-avidin (50 ng in 100 mL of 4xSSC/5% blocking reagent; Vector Laboratories, Burlingame, CA, USA), each for 30 min. Between incubations with the antibodies, the slides were washed three times in 4xSSC/0.05% Tween 20, each for 5 min. After incubations with antibodies, the slides were washed twice in 4xSSC/0.05% Tween 20 and once in 1xPBS, each for 5 min. Next, the slides were dehydrated in an ethanol series and air-dried. Dried slides were treated with 20 μL of Fluoroshield with DAPI (Vector Laboratories, Burlingame, CA, USA).

### 2.4. Microscopy and Image Analyses

All pictures were captured using an Olympus BX53 digital upright fluorescence microscope, equipped with a 21 megapixel high-resolution digital DP74 color camera (Olympus, Tokyo, Japan). The pictures from in situ hybridization experiments were merged and processed using the DP manager software (Olympus, Tokyo, Japan). Karyograms were prepared using Adobe Photoshop.

## 3. Results

### 3.1. Blaesodactylus boivini

**Figure 1:**
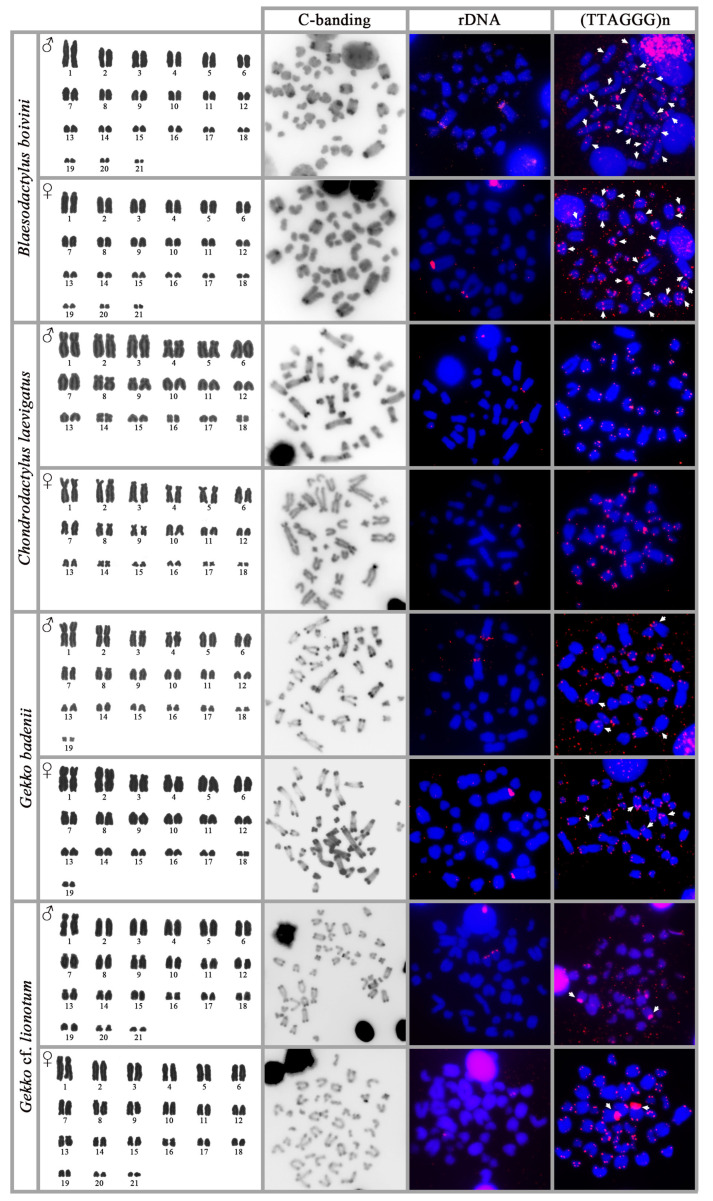
Karyograms, metaphases with C-banding stain and in situ hybridization with probes for rDNA loci and telomeric repeats from both sexes of the species *Blaesodactylus boivini*, *Chondrodactylus laevigatus*, *Gekko badenii*, and *Gekko* cf. *lionotum*. The position of interstitial telomeric repeats is indicated by arrows.

Both sexes have the diploid chromosome number 2n = 42. The karyotype is composed of only acrocentric chromosomes, gradually decreasing in size. C-banding revealed the presence of heterochromatic blocks in the centromeric regions of the six largest acrocentric pairs. rDNA loci were detected in the pericentromeric region of chromosome pair one. Telomeric repeats are present at the terminal positions of all the chromosomes and at the interstitial positions of ten chromosome pairs (Figure 1).

### 3.2. Chondrodactylus laevigatus

Both sexes present the diploid chromosome number 2n = 36. The karyotype is composed of 20 bi-armed chromosomes and 16 acrocentric chromosomes. C-banding revealed the presence of heterochromatin accumulation at the centromeres and telomeres of almost all the chromosomes. Additional heterochromatic blocks are visible in the p-arm of chromosomes from pairs one and three. rDNA probe loci are located on the chromosomes of pair five or six. Both pairs are acrocentric and equal in size; therefore, we cannot safely identify the pair with rDNA loci. Telomeric repeats are present only at the terminal positions of all the chromosomes (Figure 1).

### 3.3. Gekko badenii

Both sexes present the diploid chromosome number 2n = 38. The karyotype consists of 20 bi-armed and 18 acrocentric chromosomes. C-banding revealed the presence of heterochromatin accumulation at the centromeric and telomeric positions of almost all chromosomes. rDNA loci are located at a secondary constriction on chromosome pair one. Telomeric repeats are present at the terminal positions of all the chromosomes. Additionally, accumulation of telomeric repeats has been detected at the centromeres of chromosome pair three or four and at the interstitial positions of chromosome five or six. Chromosome pairs three and four and the chromosome pairs five and six have similar morphology, and we are not able to accurately distinguish them (Figure 1).

### 3.4. Gekko cf. lionotum

Both sexes present the diploid chromosome number 2n = 42. The karyotype consists of 12 bi-armed and 30 acrocentric chromosomes. C-banding revealed the presence of heterochromatin accumulation at the centromeric and telomeric positions of almost all the chromosomes. rDNA loci are located in a medium-sized pair of acrocentric chromosomes. Telomeric repeats are present at the terminal positions on all the chromosomes; additionally, a big block of telomeric repeats accumulates in the centromere of chromosome pairs two or three (Figure 1).

### 3.5. Hemidactylus sahgali

**Figure 2:**
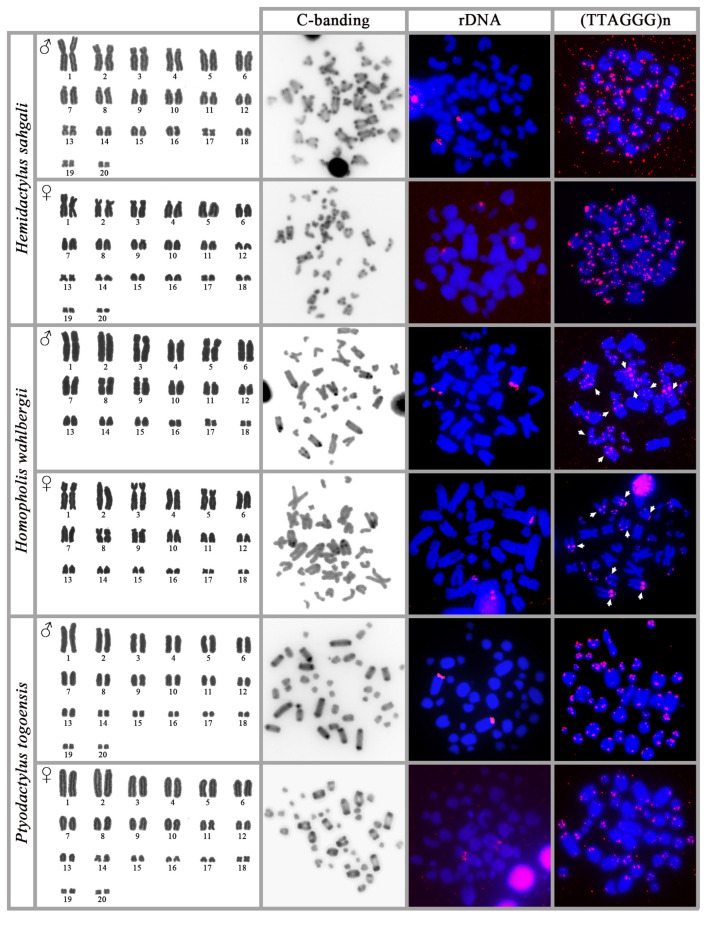
Karyograms, metaphases with C-banding stain and in situ hybridization with probes for rDNA loci and telomeric repeats from both sexes of the species *Hemidactylus sahgali*, *Homopholis wahlbergii*, and *Ptyodactylus togoensis*. The position of the interstitial telomeric repeats is indicated by arrows.

Both sexes present the diploid chromosome number 2n = 40. The karyotype consists of 20 bi-armed and 20 acrocentric chromosomes. C-banding revealed the presence of heterochromatin in the centromeric and telomeric regions of all chromosomes. rDNA loci are located on a pair of big acrocentric chromosomes. Telomeric repeats are present at the telomeric positions of all the chromosomes and at the interstitial position in a pair of medium sized chromosomes (Figure 2).

### 3.6. Homopholis wahlbergii

Both sexes present the diploid chromosome number 2n = 36. The karyotype consists of 16 bi-armed and 20 acrocentric chromosomes. C-banding revealed weak signals of heterochromatin at centromeric positions but also heterochromatic blocks at centromeric and pericentromeric regions in chromosome pairs two, four, six, and seven. rDNA loci are located at the telomeric region of the q arm of chromosome pair one. Telomeric repeats are present at the terminal positions of all the chromosomes, but also in the pericentromeric regions of four acrocentric chromosomes, possibly matching the heterochromatic block detected by C banding (Figure 2).

### 3.7. Ptyodactylus togoensis

Both sexes present the diploid chromosome number 2n = 40. The karyotype consists of 10 small, bi-armed chromosomes and 30 acrocentric chromosomes. C-banding revealed the presence of heterochromatin in the centromeric and telomeric regions of all the chromosomes, with more intense signals in the first seven pairs. rDNA loci are located at the centromere region of chromosome pair one. Telomeric repeats are present at the terminal positions of all the chromosomes (Figure 2).

## 4. Discussion

To the best of our knowledge, we present here for the first time the karyotypes of *Blaesodactylus boivini* (2n = 42), *Chondrodactylus laevigatus* (2n = 36), *Gekko* cf. *lionotum* (2n = 42), *Hemidactylus sahgali* (2n = 40), *Homopholis wahlbergii* (2n = 36) (Gekkonidae), and *Ptyodactylus togoensis* (2n = 40) (Phyllodactylidae). The karyotype of *Gekko badenii* matches the previous report under the synonym *Gekko ulikovskii* by [10] from a male individual. All seven examined species show the expected karyotypic traits of geckos, in terms of diploid chromosome numbers and chromosome morphology. A karyotype with 2n = 38 and only acrocentric chromosomes is widely assumed to be the ancestral trait for geckos [9,29,30] while the extant species have diploid chromosome numbers mainly in the range from 2n = 36 to 2n = 42 and karyotypes with mainly acrocentric chromosomes [7,11,12,14,15,16,20,31]. In contrast to other vertebrate lineages, such as mammals [32], the sauropsids have an overall low rate of interchromosomal rearrangements [10,11,33], which can explain the relatively narrow variation of diploid chromosome numbers in geckos. Nevertheless, both interchromosomal (e.g., fusion and fission) and intrachromosomal rearrangements (inversion and centromere repositioning) do occasionally occur in geckos [10,11]. Notable examples are the increase in chromosome number from 2n = 38 to 2n = 44 caused by chromosome fissions in several species of the genus *Hemidactylus* [10] or the variability in chromosome morphology (but not diploid chromosome number) in the species of the genus *Gekko* due to pericentric inversions or centromere repositioning [11].

Another observation in the species studied by us is the limited number of microchromosomes, a common trait among geckos, in contrast to other squamates, which often show a karyotype with few large macrochromosomes and several microchromosomes [33,34,35,36,37,38,39,40,41]. For example, the putative ancestral karyotype of snakes consists of 12 large, bi-armed chromosomes and 24 microchromosomes, a trait still observed in the majority of the extant snake species [42]. Recent studies documented that the genomic regions homologous to microchromosomes in other squamates seem to form medium-sized macrochromosomes in geckos [7,40]. Despite recent advances in genomics and cytogenetics, and state of the art reconstructions [7,33,43,44,45], we cannot safely conclude if the ancestral squamate karyotype was gecko-like (i.e., mainly acrocentric chromosomes and gradually decreasing in size) or “bimodal” (i.e., mixture of macrochromosomes and microchromosomes), mainly due to the unresolved phylogenetic relationships of the geckos in comparison to dibamids and the rest of the squamates (see conflicting topologies in [4,46,47,48,49,50,51,52]).

Chromosome rearrangements can be potentially detected by the presence of interstitial telomeric repeats in the karyotype. These ITRs are either remnants of chromosome rearrangements such as fusions and inversions, which transfer directly telomeric repeats from terminal to interstitial positions or are created *de novo* at the regions of DNA chromosome breaks (e.g., caused by recombination, inversions, fusions, or mobility of transposable elements) by the DNA repair machinery [53,54,55,56,57]. In addition, telomeric-like repeats often form a part of the centromeric microsatellite content, and these are not necessarily connected to chromosome rearrangements [58]. ITRs have been detected in many vertebrate taxa, including more than 100 species of lizards, snakes, and turtles (reviewed in [59,60]), including the gekkonids *Agamura persica*, *Cyrtopodion scabrum*, *Gekko hokouensis*, *Hemidactylus platyurus*, *Paroedura bastardi*, and *Paroedura stumpffi*, and the phyllodactylid *Gymnodactylus amarali* [7,59,61]. In the current study, we identified ITRs in an additional five gekkonid species: *Blaesodactylus boivinii*, *Gekko badenii*, *Gekko* cf. *lionotum*, *Hemidactylus sahgali*, and *Homopholis wahlbergii*. The topology of ITRs differs significantly among species, e.g., even between the congeneric *Gekko badenii*, *Gekko hokouensis*, and *Gekko* cf. *lionotum*, a pattern previously observed in other reptilian lineages [59,60,61]. Comparative chromosome painting or comparison of chromosome-level genome assemblies is required to identify the origin of these ITRs.

In addition to telomeric repeats, we also examined the topology of rDNA loci as a putative marker to identify sex-specific differences (i.e., sex chromosomes) in the karyotype. Both ITRs and rDNA loci are often detected in sex chromosomes [62,63,64,65,66,67,68,69], even in systems with a low level of Y/W differentiation [38,39,70]. In fact, sex chromosomes were previously identified in the genera *Gekko* and *Hemidactylus*, with either cytogenetic or genomic approaches [10,71,72,73,74], and therefore, we expected the presence of sex chromosomes in some of the congeneric species that we studied. Our cytogenetic analysis did not reveal the presence of sex chromosomes in any of the examined species. All examined species have rDNA loci located on a pair of chromosomes in both sexes, which is the expected pattern in the majority of the studied reptiles [36,75,76,77]. The position of rDNA loci differs among species, at least as far as we are able to judge based on chromosome sizes, a pattern that has been reported previously in other geckos [7,8,10,12,16,78,79]; however, we cannot determine if these chromosomes are homologous among species or rDNA loci have been translocated to different positions during the divergence of the gekkotan lineages.

We assume that the studied species either lack sex chromosomes and sex is determined by environmental factors, or their sex chromosomes are homomorphic and poorly differentiated, below the detection efficiency of our cytogenetic methods. Despite the fact that sex chromosomes are well differentiated and stable in the long term in several geckos from the genera *Paroedura* [80] and *Uroplatus* [81], turnovers between the XX/XY and ZZ/ZW systems have been reported inside the genera *Gekko*, *Cyrtodactylus*, and *Hemidactylus* (reviewed in [21]). Notably, sex chromosome differentiation might differ between populations of the same species, as in the phyllodactylid *Thecadactylus rapicauda*, where the W chromosome is detectable by cytogenetic methods in populations from Venezuela but not from Guatemala [82]. However, ESD has been well documented in the genera *Tarentola* (Phyllodactylidae) and *Phelsuma* (Gekkonidae) [24], with unverified reports in several gekkonids. Most notable is the case of *Gekko japonicus*, where cytogenetic analysis identified a XX/XY sex determination system [74,83], but incubation of eggs in a wide range of temperatures revealed significant variation in the hatchlings’ sex ratio, with lower and higher temperatures leading to female-biased progeny and middle temperatures leading to male-biased progeny, which reveals a pattern typical for ESD [72]. We assume that *Gekko japonicus* might be a similar case to the dragon lizard *Pogona vitticeps*, where certain environmental conditions (e.g., extremely high incubation temperatures in the case of the dragon lizard) can override the effect of sex chromosomes, leading to sex reversals [84].

Overall, geckos demonstrate limited variability in karyotypic traits, including chromosome morphology and diploid chromosome numbers. On the other hand, they demonstrate extensive variability in sex determination systems. Geckos are an old radiation, and sex chromosomes might be homomorphic and hard to detect by conventional methods, which might undermine our estimations on the stability of the gekkotan sex determination systems. We propose that additional research should be conducted in the herein studied species, as well as in other unstudied geckos, using both (i) incubation experiments of eggs in a range of temperatures in laboratory conditions to examine variation in the hatchlings’ sex ratio, which can indicate the presence of an ESD or a GSD system and (ii) genomic approaches to identify the presence of sex chromosomes, which can reveal a GSD system and identify the sex chromosome gene content (e.g., whole genome sequencing—DNAseq and gene coverage analysis or SNPs analysis from Restriction site-associated DNA sequencing—RADseq).

## Figures and Tables

**Table 1:** Number of individuals per species and sex from seven species of geckos, analyzed in this study. The sex was identified by external morphology.

Species	Family	Diploid Number (2n)	Males	Females
*Blaesodactylus boivini*	Gekkonidae	42	1	1
*Chondrodactylus laevigatus*	Gekkonidae	36	2	2
*Gekko badenii*	Gekkonidae	38	2	7
*Gekko* cf. *lionotum*	Gekkonidae	42	1	1
*Hemidactylus sahgali*	Gekkonidae	40	1	1
*Homopholis wahlbergii*	Gekkonidae	36	2	2
*Ptyodactylus togoensis*	Phyllodactylidae	40	1	1

## Data Availability

Not applicable.
